# Modeling the structure of the frameshift-stimulatory pseudoknot in SARS-CoV-2 reveals multiple possible conformers

**DOI:** 10.1371/journal.pcbi.1008603

**Published:** 2021-01-19

**Authors:** Sara Ibrahim Omar, Meng Zhao, Rohith Vedhthaanth Sekar, Sahar Arbabi Moghadam, Jack A. Tuszynski, Michael T. Woodside

**Affiliations:** 1 Department of Physics, University of Alberta, Edmonton, Alberta, Canada; 2 Department of Oncology, University of Alberta, Edmonton, Alberta, Canada; University of Maryland School of Pharmacy, UNITED STATES

## Abstract

The coronavirus causing the COVID-19 pandemic, SARS-CoV-2, uses −1 programmed ribosomal frameshifting (−1 PRF) to control the relative expression of viral proteins. As modulating −1 PRF can inhibit viral replication, the RNA pseudoknot stimulating −1 PRF may be a fruitful target for therapeutics treating COVID-19. We modeled the unusual 3-stem structure of the stimulatory pseudoknot of SARS-CoV-2 computationally, using multiple blind structural prediction tools followed by μs-long molecular dynamics simulations. The results were compared for consistency with nuclease-protection assays and single-molecule force spectroscopy measurements of the SARS-CoV-1 pseudoknot, to determine the most likely conformations. We found several possible conformations for the SARS-CoV-2 pseudoknot, all having an extended stem 3 but with different packing of stems 1 and 2. Several conformations featured rarely-seen threading of a single strand through junctions formed between two helices. These structural models may help interpret future experiments and support efforts to discover ligands inhibiting −1 PRF in SARS-CoV-2.

## Author summary

The coronavirus that causes COVID-19 controls the production of key viral proteins through a process known as programmed ribosomal frameshifting. Frameshifting is triggered by a particular structure in the viral RNA, a pseudoknot, which is a promising drug target. Here we model the structure of this pseudoknot through atomistic molecular dynamics simulations. Surprisingly, we find that the pseudoknot can take on distinct fold topologies, two of which involve unusual threading of a single strand of RNA through helical junctions, something not seen before in frameshifting pseudoknots. All of the folds are generally consistent with previous experimental studies of the closely-related SARS coronavirus pseudoknot. These results should assist in the analysis and interpretation of future experimental studies of the pseudoknot structure, and support structure-based drug-discovery efforts.

## Introduction

The COVID-19 pandemic caused by the novel Severe Acute Respiratory Syndrome coronavirus 2 (SARS-CoV-2) has spread across the globe since the virus emerged in late 2019 [1]. Given the high infectivity of SARS-CoV-2 and the novel immunological challenge it poses to human hosts, epidemiological modeling suggests that recurring outbreaks with elevated mortality can be expected even despite successful public-health responses, until vaccines or preventive drugs can be found to inhibit transmission [2]. The discovery of effective treatment therapeutics is thus one of the central goals of research into COVID-19 [3].

One potential target for treatment is the frameshift-stimulatory pseudoknot found between the overlapping ORF1a and ORF1b in the SARS-CoV-2 genome [4]. Like other human coronaviruses [5], SARS-CoV-2 depends on −1 programmed ribosomal frameshifting (−1 PRF) to produce essential proteins at regulated levels [6]. In −1 PRF, a shift in reading frame is stimulated at a specific location in the RNA message by a structure in the mRNA—typically a pseudoknot—located 5–7 nucleotides downstream of a ‘slippery’ sequence, thereby generating more than 1 protein from the same message [7,8]. The level of frameshifted gene products must often be held within a tight range for optimal propagation of the virus, hence disrupting −1 PRF by modulating the efficiency of frameshifting can attenuate the virus. Indeed, inhibiting −1 PRF was found to suppress replication of the close relative SARS-CoV-1 by orders of magnitude [9,10], suggesting that the same strategy may be effective against SARS-CoV-2.

**Fig 1:**
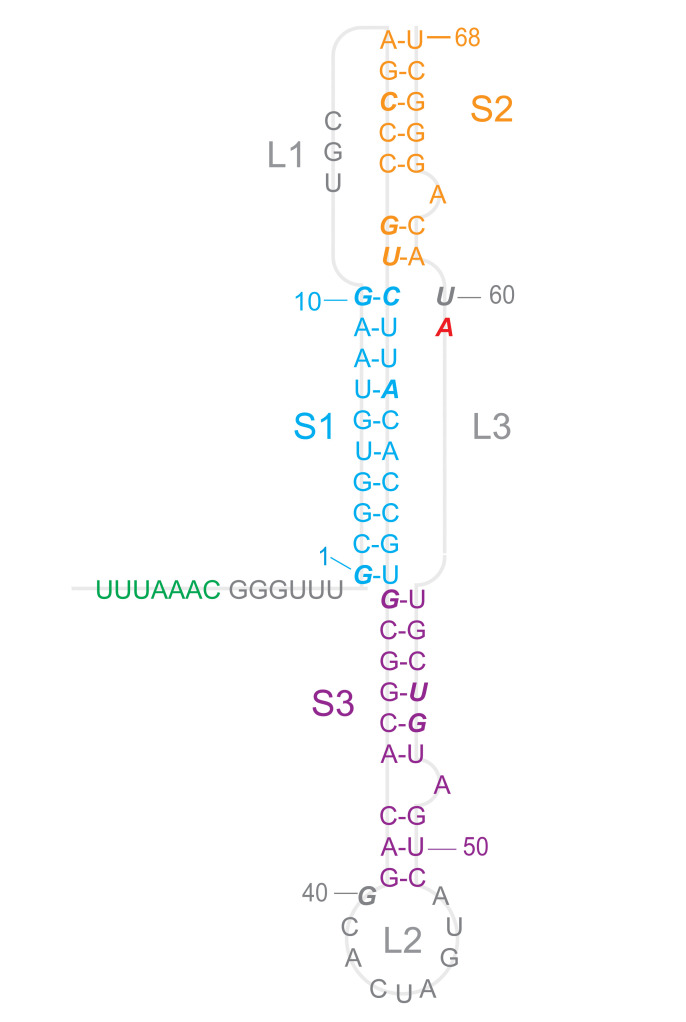
SARS-CoV-2 pseudoknot primary and secondary structure. The sequence is color-coded by secondary structure (S1: cyan, S2: orange, S3: purple, loops: grey). The only difference from SARS-CoV-1 is that A59 (red) is changed from C59 in the latter. Bases shown in italic are protected against nuclease digestion in SARS-CoV-1. Slippery sequence shown in green.

The structure of the frameshift-stimulatory pseudoknot has not yet been solved at high resolution for either SARS-CoV-1 or SARS-CoV-2, however, hindering structure-based drug-discovery efforts. The primary sequences of these two pseudoknots are almost identical, differing by a single nucleotide, hence their secondary structure is expected to be the same. Evidence from computational methods, nuclease protection assays, and 2D NMR spectroscopy applied to the SARS-CoV-1 pseudoknot [11,12] indicates it has a 3-stem architecture that is unusual for frameshift-stimulatory pseudoknots: whereas such pseudoknots typically consist of 2 interleaved stems and loops, here the second loop is greatly extended and a third stem-loop combination forms within it (Fig 1). Bulged adenine residues in stem 2 (S2) and stem 3 (S3) seem to play important functional roles, as mutating them to cytosine abolished or reduced −1 PRF (respectively for the bulges in S2 and S3) [12]. Cryo-electron microscopy has been used to model the SARS-CoV-2 pseudoknot structure on its own [13] and on the ribosome [14], but with conflicting results; the full 3D structure has not yet been solved for any other 3-stem pseudoknot.

Computational modeling provides an alternative approach to characterizing the structure of these pseudoknots, but such modeling has been limited to date. One study assembled a 3D structure of the SARS-CoV-1 pseudoknot by hand with the Sybyl chemical modeling package before equilibrating briefly with 1 ns of molecular dynamics (MD) simulation [15], and another study used the Rosetta FARFAR2 platform [16,17] to make a ‘blind’ prediction of the structure of the SARS-CoV-2 pseudoknot [18]. Here, we have modeled the structure of the SARS-CoV-2 pseudoknot more extensively, using blind predictions from multiple platforms as inputs for μs-long MD simulations to examine the stability of the structures. We also assessed the ensemble of structures observed in the simulations for their consistency with previous work on the biochemical and biophysical properties of the SARS-CoV-1 pseudoknot to identify the most likely structural models. We found several possibilities, all sharing an extended S3 helix but differing in the S1/S2 packing and junction with S3.

## Methods

### Blind structure prediction

Initial structures for input into MD simulations of the monomeric pseudoknot were obtained using multiple platforms for blind RNA structure prediction: SimRNA [19], Rosetta FARFAR2 [16,17], RNAComposer [20], RNAvista [21], MC-Sym [22], RNA2D3D [23], and Vfold [24]. For blind predictions, we assumed the secondary structure shown in Fig 1, based on previous characterization of the secondary structure of the SARS-CoV-1 pseudoknot [12]. Blind predictions of pseudoknot dimer structures were made using FARFAR2, which allows for dimer structure prediction, or constructed manually using the Molecular Operating Environment software based on monomeric models from other prediction platforms, which do not directly allow for dimer predictions.

### MD simulations

Models from blind structure predictions were used as starting structures for all-atom MD simulations in explicit solvent using Amber 18 [25]. The models were protonated at pH 7 using Molecular Operating Environment. The pseudoknots were parameterized using the f99bsc0_chiOL3 force-field and were solvated in optimal point charge water boxes with minimum margins of 12 Å using the tleap module of Amber. The solvated systems were first neutralized using sodium ions, then their salinities were adjusted to 0.15 M NaCl using Joung-Cheatham monovalent ion parameters [26]. Each pseudoknot model was simulated under two conditions: without Mg^2+^ ions, or with six Mg^2+^ ions placed initially at the junction between S1 and S3 as well as along the backbone of S2. The solvated systems were energy-minimized then heated to 310 K with heavy restraints of 10 kcal/mol/A^2^ on the backbone phosphate atoms. These restraints were gradually removed and the unrestrained systems were then simulated on graphical processing units for 1 μs at constant pressure.

### Analysis of simulated models

Analysis was performed using the CPPTRAJ module of AmberTools [25]. Different conformations of the pseudoknot within each simulation were clustered based on the root-mean-squared deviation (RMSD) of residues G1–G40 and C49–G66 (omitting the residues in L2 and at the 3′ terminus, which tended to have large fluctuations), using the hierarchical agglomerative approach. The representative structures of the three most populated clusters of each model (S1 Data) were visually assessed for helical distortions in S1, S2 and S3. The hydrogen bonding of the residues identified as protected in SARS-CoV-1 by nuclease-protection assays [12] as well as of bulged adenines and residues in L1 were also calculated, reporting the interactions formed by hydrogen bond donors and acceptors between two bases or between a base and the backbone atoms of other residues that were present for at least the last 100 ns of the trajectory. The root-mean-squared fluctuation (RMSF) of each residue was also calculated.

## Results

**Fig 2:**
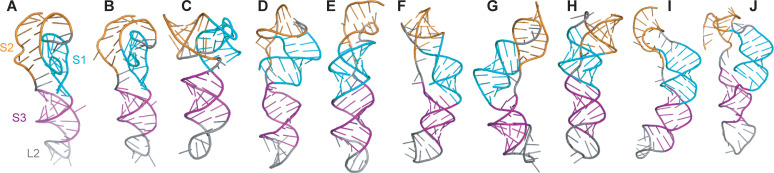
Blind predictions of pseudoknot structure. Structures predicted by (A) RNAvista, (B) RNAComposer, (C–E) SimRNA, (F–H) Rosetta FARFAR2, (I, J) Vfold. In each case, secondary structure is color-coded (S1: cyan, S2: orange, S3: purple, loops: grey).

We made initial estimates of the pseudoknot structure using a variety of tools that have been developed for blind prediction of RNA structure [16,17,19–21,23], where the only input was the sequence and the expected secondary structure, as shown in Fig 1. Some of these predictions were rejected as being implausible because they contained topological knots, which cannot occur but were seen in predictions from MC-Sym, RNAvista, and RNA2D3D (S1 Table). The remainder are illustrated in Fig 2; note that those in Fig 2F–2H were also reported previously in a separate study [18]. These predictions were quite varied. They all showed S3 as an extended helix lacking obvious contacts with the rest of pseudoknot except right near the junction with S1. However, the arrangement of S1 and S2 and L1 and L3 differed significantly between the predictions. In several models (Fig 2A–2D), S1 and S2 were packed very tightly, leading to distorted or broken base-pairs in order to accommodate the packing. A few models (Fig 2E, 2G, and 2J) showed an unusual quasi-knotted structure where the 5′ end was threaded through the junction between S1 and S3, a fold topology that has only been seen previously in exoribonuclease-resistant RNAs [27]. Another model (Fig 2H) showed a similar knot-like topology, but this time with L3 (near the 3′ end) threaded through the junction between S1 and S2. Note that in several cases, the first three nucleotides upstream of the 5′ end of the pseudoknot (UUU) were included in the modeling, in order to distinguish the possibilities for 5′-end threading.

To examine if the blind predictions were dynamically stable, we used them to initiate extended all-atom molecular dynamics simulations. Each structure in Fig 2 was simulated for at least 1 μs in explicit solvent under two conditions: with NaCl only, or with both NaCl and Mg^2+^ ions. Both conditions were used because not all pseudoknots require Mg^2+^ ions to fold [28,29], and it is unclear if Mg^2+^ ions are essential for the SARS-CoV-2 pseudoknot. The first part of the simulation was treated as an equilibration phase and only the last 500 ns of the simulation was examined in each case. Because the simulations were dynamic, we clustered the structures occupied in the simulations by RMSD and examined the centroid (representative) structures of the three most occupied clusters.

**Fig 3:**
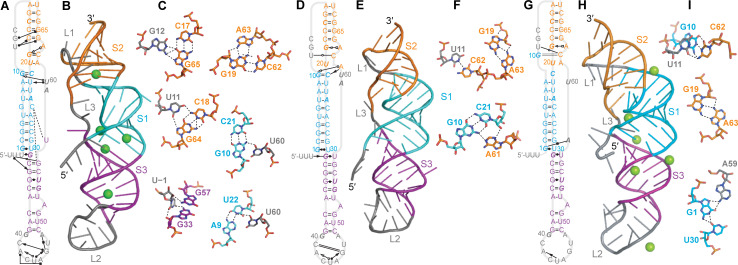
Representative structures from MD simulations of unthreaded models. (A–C) Structure from simulation of Fig 2F with Mg^2+^. (A) Secondary structure with tertiary contacts. Interactions indicated with the notation from Ref. (30). Bases shown in italic are protected against nuclease digestion in SARS-CoV-1. (B) Representative 3D structure of most populated cluster. Green spheres: Mg^2+^ ions. (C) Close-up view of key tertiary contacts. (D–F) Same for simulation of Fig 2F without Mg^2+^, showing fewer tertiary contacts than with Mg^2+^ in (A). (G–I) Same for simulation of Fig 2I with Mg^2+^.

**Fig 4:**
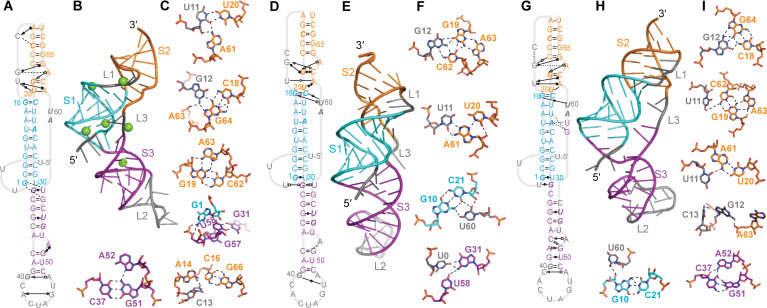
Representative structures from MD simulations of models with 5′-end threading. (A–C) Structure from simulation of Fig 2G with Mg^2+^. (A) Secondary structure with tertiary contacts, notated as in Fig 3. (B) Representative 3D structure of most populated cluster. Mg^2+^ ions shown in green. (C) Close-up view of key tertiary contacts. (D–F) Same for simulation of Fig 2E without Mg^2+^. (G–I) Same for simulation of Fig 2G without Mg^2+^ showing opening of the top of S3.

**Fig 5:**
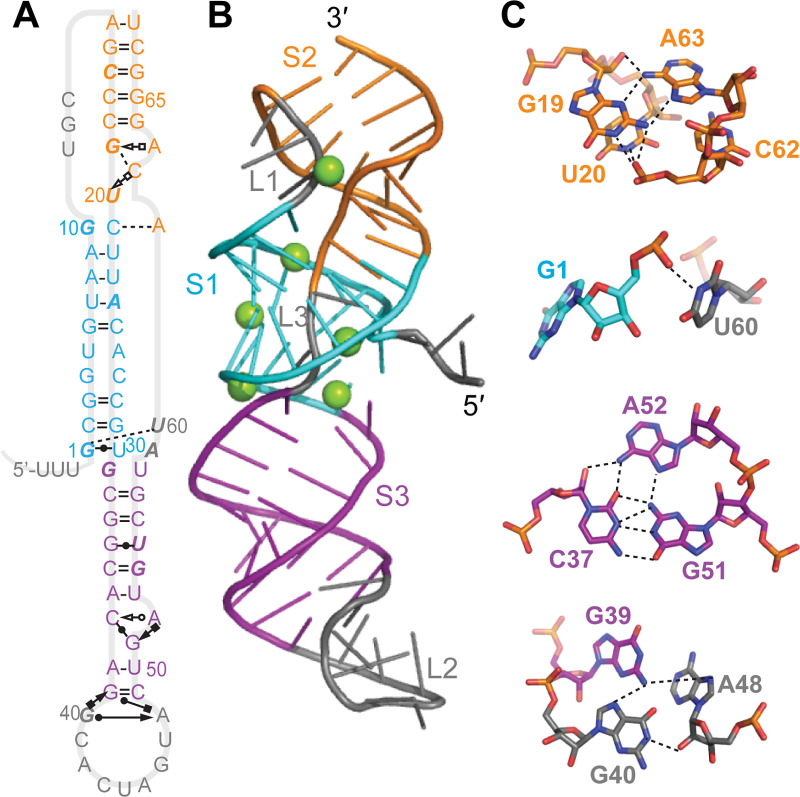
Representative structures from MD simulations of models with L3 threading. (A–C) Structure from simulation of Fig 2H with Mg^2+^. (A) Secondary structure with tertiary contacts, notated as in Fig 3. Bases shown in italic are protected against nuclease digestion in SARS-CoV-1. (B) Representative 3D structure of most populated cluster. Mg^2+^ ions shown in green. (C) Close-up view of key tertiary contacts.

Several of the initial models led, after equilibration in MD simulations, to structures that featured various combinations of significant defects in the expected base-pairing for S1, defects in S2, and/or a lack of tertiary contacts (examples shown in S1 Fig). In some cases, these structures were sufficiently unstable that they unfolded substantially. Any model containing more than one broken base-pair in a given stem without the un-pairing being compensated by alternative interactions was therefore rejected as unlikely to be correct (model rejections listed in S1 Table). The other initial models yielded structures under at least one of the MD simulation conditions that were more plausible, and they were thus analyzed in more detail. The results could be arranged into three groups: structures without any threading at either end from Rosetta FARFAR2 and Vfold (Fig 3), structures with the 5′ end threaded through the S1/S3 junction from FARFAR2 and SimRNA (Fig 4), and a structure with L3 threaded through the S1/S2 junction from FARFAR2 (Fig 5).

Considering first the structures that were more similar to standard H-type pseudoknots, without any threading of either end through stem junctions, the representative structures of the most populated clusters with and without Mg^2+^ are shown in Fig 3, indicating the canonical and non-canonical interactions with standard notation [30]. From the simulations of the FARFAR2 model with Mg^2+^ (Fig 3A–3C), several triples and triple-like interactions were identified in S2/L1 (Fig 3A), and the Watson-Crick edge of U60 in L3 was seen to interact with the sugar edges of both G10 and U22 in S1. Base-pairs in S1 (G6:C25) and S3 (G31:U58) were disrupted, in favor of base-backbone bonding between C25 and both G57 and U58, and a wobble pair with the end of the spacer (G31:U0). A network of non-canonical base-pairs was also seen in L2. The stabilization from the hydrogen-bond networks led to low fluctuations, even in L2, and the RNA spent almost all of its time in the top 3 clusters (S2A Fig). There was no stacking between S1 and S2, but G31:U0 stacked on S1. For the simulations of the FARFAR2 model without Mg^2+^ (Fig 3D–3F), the disrupted base-pairs in S1 and S3 were restored, but the triples in S2/L1 were absent and the pairing in the lower part of S2 was disrupted in favor of non-canonical interactions between U11:C62 and G19:A63. However, the sparser network of tertiary interactions led to higher fluctuations and a greater diversity of clusters than with Mg^2+^ present (S2B Fig). There was no stacking of S1 with S2 or S3 in this structure. Finally, in the simulations of the Vfold model with Mg^2+^ (Fig 3G–3I), the G10:C21 pair in S1 was disrupted in favor of interactions with C62 in S2, which also interacted with U11. Non-canonical interactions were again formed between G19 and A63, but the sparse network of tertiary interactions led to relatively high fluctuations, even though the RNA spent almost all of its time in the top 3 clusters (S2C Fig). As with Fig 3A, there was no stacking between S1 and S2, but G31:U58 at the end of S3 stacked on S1.

Turning next to the representative structures of the most populated clusters with the 5′ end threaded through the S1/S3 junction, we again found three possibilities. In the simulations of the FARFAR2 model with Mg^2+^ present (Fig 4A–4C), several triples/triple-like interactions were identified in S2/L1, two of them the same as in Fig 3A–3C. The opening G:U pair in S1 was disrupted in favor of interactions with G57 and U58, to accommodate the threading of the 5′ end through the S1/S3 junction, and S3 was extended by a non-canonical pair between G40 and A48. L2 was again structured by a network of hydrogen bonds, but L3 did not interact with any other part of the structure other than a coordinated Mg^2+^ ion. In the simulations of the SimRNA model without Mg^2+^ (Fig 4D–4F), the 5′ end was threaded through the S1/S3 junction without disrupting the base-pairing near the junction, stabilized by interactions between the Watson-Crick edge of G31 and the Hoogsteen edge of U0. Only one triple was seen in S2/L1, but it also interacted with the Hoogsteen edge of the bulged A63 in S2. The U20:A61 pair at one end of S2 was distorted to include interactions between U11 and A61, and U60 in L3 was bonded to both G10 and C21, analogous to the situation in Fig 3A–3C. L2 was partially structured by interactions between G40 and U47. Finally, in the simulations of the FARFAR2 model without Mg^2+^ (Fig 4G–4I), the first two base-pairs in S3 next to the S1/S3 junction were opened to facilitate the 5′-end threading, stabilized by a U0:G31 wobble pair, with the unpaired G57 and U58 forming H-bonds with the backbone at A24 and U23. Triples/triple-like interactions in S2 analogous to some of those seen in Figs 3A–3C and 4A–4C were present, and once again U60 in L3 interacted with G10 in S1 through the sugar edges. The bottom of S3 was also re-arranged, forming a triple with the bulged A52 and extending the stem with the pair G40:C49 and non-canonical bonding between C41 and A48. As with the unthreaded structures, the simulation with Mg^2+^ showed smaller fluctuations than those without Mg^2+^; the fraction of the trajectory spent in the top 3 clusters was very high (over 90%) for the simulation with Mg^2+^ and the first model without Mg^2+^, but lower (66%) for the second model without Mg^2+^ (S3 Fig). Unlike in the unthreaded structures, however, S1 and S2 were stacked in all three 5′-threaded structures, whereas S1 and S3 were unstacked.

Considering the representative structure of the most populated cluster for the third fold topology, with L3 threaded through the S1/S2 junction, only the result with Mg^2+^ is shown (Fig 5A–5C), as the structure was unstable without Mg^2+^. Here, the threading of L3 disrupted base-pairing in the bottom of S2 and the middle of S1, although the two strands of S2 continued to interact via base-backbone hydrogen bonds and S1 still retained a fairly regular helical structure; U60 bonded with the backbone of G1 to help stabilize the threading of L3. In S3, the same triple formed as in Fig 4G–4I, but without the re-configuration and extension of the stem. L2 was partially structured by base-backbone bonds between G40, A48, and the closing base-pair of S3. The threading of L3 prevented any stacking of the helices. The top three clusters comprised 78% of the MD trajectory, with relatively low fluctuations (S4 Fig).

**Fig 6:**
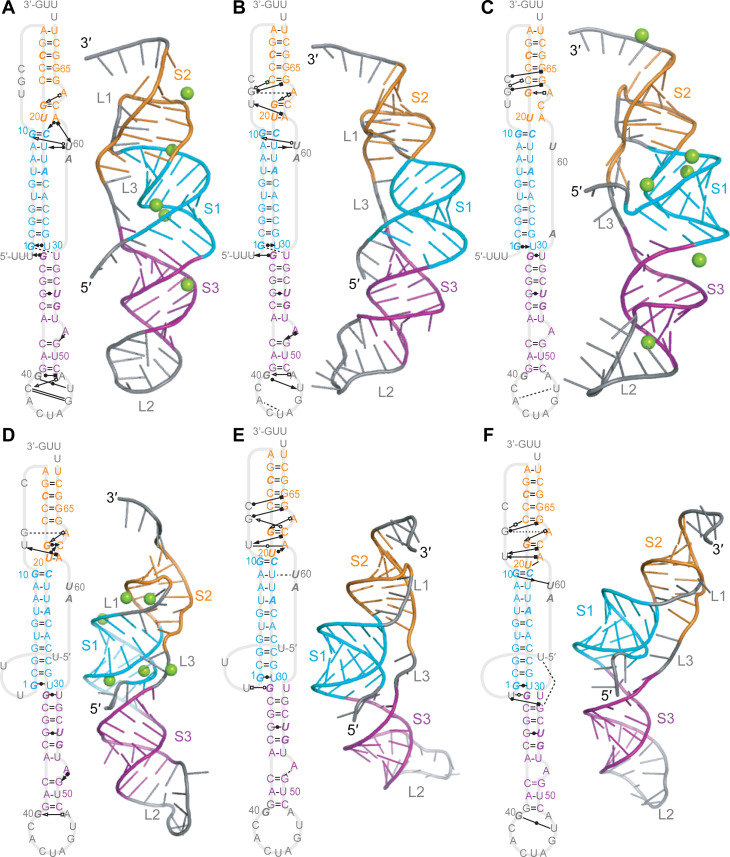
Representative structures from simulations with extended 3′ ends. Secondary structures and representative 3D structures of the most populated clusters. (A) Unthreaded model from FARFAR2 with Mg^2+^. (B) Unthreaded model from FARFAR2 without Mg^2+^. (C) Unthreaded model from Vfold with Mg^2+^. (D) 5′-threaded model from FARFAR2 with Mg^2+^. (E) 5′-threaded model from SimRNA without Mg^2+^. (F) 5′-threaded model from FARFAR2 without Mg^2+^.

Because it is unclear if the nucleotides downstream of the pseudoknot might affect the stability of these structures, we repeated the simulations of each of the models shown in Figs 3–5 while extending the pseudoknot at the 3′ end by the next 4 nucleotides (UUUG). For the unthreaded and 5′-threaded models from Figs 3 and 4 (Fig 6), the network of tertiary interactions in L1 and S2 became more dense in half of the structures (Fig 6B, 6C, and 6E), in one it was little changed (Fig 6F), and in two of the structures with Mg^2+^ it became more sparse (Fig 6A and 6D). The network of interactions in L2 also showed changes. Helix stacking was generally unchanged, except that S1 and S2 became stacked in the unthreaded model without Mg^2+^ (Fig 6B), whereas they became unstacked in the 5′-threaded model with Mg^2+^ (Fig 6C). In contrast to these modest changes, however, half of S1 unfolded with the additional nucleotides present on the 3′ end for the L3-threaded model from Fig 5, suggesting that this fold topology is not as stable as the others.

**Fig 7:**
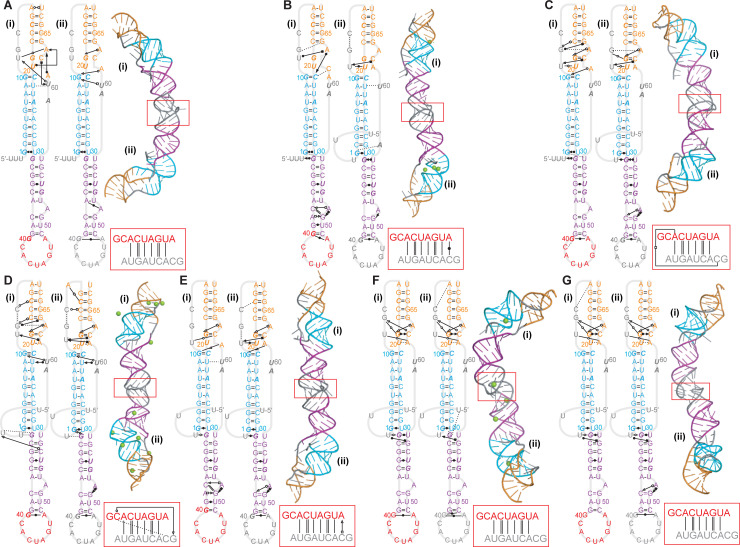
Representative dimer structures from MD simulations. Secondary structures and representative 3D structures of the most populated clusters for the models consistent with experimental data. (A) Unthreaded/unthreaded dimer without Mg^2+^. (B) Unthreaded/5′-threaded dimer with Mg^2+^. (C) Unthreaded/5′-threaded dimer without Mg^2+^. (D, E) 5′-threaded/5′-threaded dimer from FARFAR2 with (D) and without (E) Mg^2+^. (F, G) 5′-threaded/5′-threaded dimer from SimRNA with (F) and without (G) Mg^2+^. In each case, the interactions in the dimerization domain are shown in the red box.

Finally, the pseudoknot for SARS-CoV-1 is known to dimerize through a palindromic sequence in L2, and dimerization modulates the stimulation of −1 PRF [31]. We therefore simulated dimeric pseudoknots to explore if the structures from the monomeric models were altered significantly upon dimerization. Five dimer predictions from Rosetta FARFAR2 with different combinations of fold topologies (unthreaded/unthreaded, unthreaded/5′-threaded, unthreaded/3′-threaded, 5′-threaded/3′-threaded, and 5′-threaded/5′-threaded) as well as one dimer constructed from the 5′-threaded prediction of SimRNA (Fig 2E) were simulated for 1 μs both with and without Mg^2+^, as for the monomers. All the models containing 3′-end threading showed significant unfolding of S1 and/or S2 in the MD simulations and were therefore rejected as inconsistent with the known base-pairing; so too was the unthreaded/unthreaded dimer with Mg^2+^, in which almost half of S2 was unpaired (S1 Table). However, the other combinations of folds were all stable with and/or without Mg^2+^ present (Fig 7).

Considering the unthreaded/unthreaded dimer, only the structure without Mg^2+^ was stable (Fig 7A). Domain (i) in this dimer had more extensive tertiary contacts in L1, S2, and U60 from L3 than domain (ii) (or than the comparable monomeric structure, Fig 3D–3E), leading to tighter packing of S1/S2, but none of the helices were stacked. The unthreaded/5′-threaded dimer was stable both with and without Mg^2+^ (Fig 7B and 7C). However, the tertiary interactions in L1/S2 were significantly more extensive in the absence of Mg^2+^: these interactions were much reduced in the presence of Mg^2+^ for both the unthreaded and threaded domain, compared to the analogous monomeric models (respectively Figs 3A and 4A), whereas they were notably increased for the unthreaded domain in the absence of Mg^2+^, compared to the analogous monomeric model (Fig 3B). The G31:U58 wobble pair in the unthreaded domain was also broken, in both conditions. Helix stacking was the same in both conditions: S1 and S2 were not stacked in either domain, whereas S1 and S3 were stacked in the threaded domain and U0:G31 stacked on S1 in the unthreaded domain. Turning to the 5′-threaded/5′-threaded dimer from FARFAR2 (Fig 7D and 7E), it was stable in both ionic conditions, but this time the tertiary interactions in L1/S2 and interactions of U60 with S1 were significantly more extensive with Mg^2+^ present. Indeed, the interactions in the presence of Mg^2+^ were more extensive than in the analogous monomer model with Mg^2+^ (Fig 4A), whereas the interactions in the absence of Mg^2+^ were considerably less extensive than in the analogous monomer model without Mg^2+^ (Fig 4G). The stacking was also Mg^2+^-dependent: with Mg^2+^, S1 and S2 were stacked but S1 and S3 were not, whereas S1 and S2 were unstacked but U0:G31 stacked on S1 without Mg^2+^. Finally, the 5′-threaded/5′-threaded dimer from SimRNA (Fig 7F and 7G) gave quite similar results both with and without Mg^2+^, with tertiary interactions that were not much changed from the analogous monomer model without Mg^2+^ (Fig 4), although the dimer lacked any interactions with U60 as well as S1/S2 stacking, showing S1 and S3 stacked instead. Note that in all the dimer models, almost none of the tertiary interactions involving L2 seen in the monomer models were present, because many of the bases in L2 were engaged in dimerization contacts (Fig 7, red boxes). The dimerization of L2 led to only minor changes in S3 and the S1/S3 junction when compared to the analogous monomer domains, however, most notably the breaking of the G31:U58 wobble pair in several of the dimer domains, the addition or removal of one or two tertiary interactions stabilizing the S1/S3 junction, and in some cases changes to tertiary interactions involving the bulged A52.

## Discussion

Perhaps the most notable aspect of this work is that we found at least two distinct fold topologies that were persistent in long MD simulations under at least one ionic condition. Such a result contrasts with previous work simulating the pseudoknot from SARS-CoV-1, which reported only a single structure that was somewhat similar to the models with 5′-end threading, although the additional nucleotides completing the threading were not included in that model [15]. This difference can be explained by the fact that only a single initial structure was explored in that work: the distinct fold topologies we observed are sufficiently different that they cannot interconvert without substantial unfolding of the S1/S2 region, and they are sufficiently stable that such unfolding is unlikely (with the exception of the L3-threaded fold). Furthermore, the existence of multiple structures has been seen previously in various frameshift signals [32–37]. Indeed, it is entirely consistent with the hypothesis [35] that high-efficiency stimulatory structures such as that from SARS-CoV-2—which induces −1 PRF at a rate of ~20–35% [6,38]—have high conformational heterogeneity and hence form more than one structure.

The models presented above are generally consistent with previous experimental characterizations of the SARS-CoV-1 pseudoknot. Single-molecule force spectroscopy of pseudoknot unfolding for SARS-CoV-1 found a broad unfolding force distribution with high forces in the range 20–60 pN, indicating the presence of significant tertiary contact formation [35]. Consistent with this observation, all of the models feature tertiary contacts stabilizing the 3D structure, although some of the unthreaded models showed fewer such contacts, especially in S2. The unfolding of the L3-threaded fold in several of the simulations, however, suggests that this model may not be able to support the high unfolding forces observed. Turning to the results from nuclease-protection and mutation experiments [12], the different models contain features that may be matched to protected residues, such as triples, hydrogen-bond networks, or steric protection; the participation of A63 in S2 in triples is also consistent with work showing that mutating A63 can dramatically lower the −1 PRF efficiency [12]. Some of the unthreaded models without Mg^2+^ are most lacking in these features, suggesting that they may be less consistent with the experimental data. We note, however, that none of the structural models provided an obvious explanation for a few of the protected nucleotides, such as G54 and U55.

It is still unknown if Mg^2+^ is essential for the folding of this pseudoknot. Our modelling, however, suggests that Mg^2+^ helps to stabilize the structures. In every case, the fluctuations were reduced with Mg^2+^ present, and in most cases the presence of Mg^2+^ stimulated a denser network of hydrogen bonds. The role of Mg^2+^ was particularly important for threading L3 through the S1/S2 junction (Fig 5): Mg^2+^ was found to be essential for maintaining the integrity of S1, as the tight packing of the backbone that was needed could not be accommodated without the countervailing charge from the ions. Even with Mg^2+^, however, S1 and/or S2 still unfolded partially in many of the simulations, suggesting that the L3-threaded fold is not stable except possibly at high Mg^2+^ concentration.

The threaded fold topologies are particularly interesting: although they have been observed in exoribonuclease-resistant RNAs, where the 5′ end is threaded through a ring closed by a pseudoknot [27], no such fold has been seen in other frameshift-stimulatory structures. We note that threading of either the 5′ end (as in Fig 4) or L3 (as in Fig 5) requires that the different parts of the structure fold in a specific order. For example, S2 would likely need to form last for 5′-end threading, else the RNA upstream of the pseudoknot would be unable to thread through the S1/S3 junction, whereas S1 would likely need to form last for L3 threading, else the downstream RNA would be unable to thread through the S1/S2 junction. As a result, the pseudoknot would be expected to populate multiple conformers, dictated by the kinetic partitioning between the different possible pathways [39]. Different fold topologies at the 5′ end may also have important functional implications: 5′-end threading reduces the effective length of the spacer between the slippery site and the pseudoknot from 6 nt to 5 nt by sequestering U0 in the pseudoknot structure, and −1 PRF levels are quite sensitive to the spacer length [7,8,40]. The different folds might thus be expected to have different effectiveness at stimulating −1 PRF owing to the change in spacer length, in addition to any differences arising from the fold.

Although reliably evaluating the relative stabilities of the different structural models from the simulations is difficult, it is clear that the L3-threaded structure is least stable, as it is the most likely to unfold in μs-long simulations. We therefore expect that it is the least likely fold to be formed by the pseudoknot. The models that are most like other frameshift-stimulatory pseudoknots are those with 5′-end threading: despite the unusual fold topology, these are the only models that consistently show both S1/S2 stacking—which is known to play an important role in helping pseudoknots stimulate −1 PRF [41]—and dense networks of tertiary contacts, whether in monomers or dimers. The unthreaded models, in contrast, tend to have fewer tertiary contacts and little or no tendency to stack S1 and S2. As a result, we expect that the 5′-threaded fold is likely the most stable and hence dominant form of this pseudoknot. In fact, recent cryo-electron microscopy imaging of the SARS-CoV-2 pseudoknot on and off the ribosome shows evidence for 5′-end threading [13,14], although the structural models inferred from these studies differ from each other and from the models presented here, likely because of the different measurement conditions (high Mg^2+^ concentration or presence of ribosomes). Some images also suggest the presence of other conformers, consistent with the heterogeneity found in the simulations. Notably, we would expect that even if the 5′-threaded fold is the dominant conformer, unthreaded conformers will always be present, too, because the kinetic partitioning of S2 folding before or after the 5′ end is threaded into the S1/S3 junction will inevitably lead to a population of unthreaded conformers. Such an effect has been seen directly in the folding of an exoribonuclease-resistant RNA from Zika virus [27], where both threaded and unthreaded conformers were observed [42].

The structural models described above will be helpful for future experimental analyses of the SARS-CoV-2 pseudoknot. X-ray scattering profiles can be predicted from these models and used to analyze small- and wide-angle x-ray scattering measurements, to confirm which (if any) of these conformations are populated and in what kind of mixture [6,43,44]. The models could also be compared to single-molecule measurements of pseudoknot folding, which could detect heterogeneous populations of different conformers and characterize the sequence of intermediate states formed during the folding of each one [45,46], again verifying which of the conformers fold and quantifying their relative abundance and stability. These models should also prove useful for drug discovery efforts, facilitating structure-based searches for compounds that attenuate the virus by altering −1 PRF.

## Supporting information

S1 Table Pseudoknot models studied. Models in red were rejected after MD simulations because of unfolded base-pairs that were inconsistent with the expected secondary structure. Models in blue were rejected before MD simulation because of a topologically knotted fold that is inconsistent with RNA. Models in green were analyzed in the main text.
(DOCX)

S1 Fig Selected structures from simulations with significant secondary structure disruption. Representative structures of the most populated cluster from simulations of (A) Fig 2A, (B) Fig 2B, (C) Fig 2C, and (D) Fig 2D show significant disruption of the secondary structure. In each panel, the figure on the right is from simulations with Mg^2+^, that on the left is from simulations without Mg^2+^.
(TIF)

S2 Fig MD simulations of unthreaded models. (A) Overlay of the 3D structure of the 3 most populated clusters from simulations of Fig 2F with Mg^2+^ (ions not shown for clarity). Top inset: RMSD vs time, showing when each of the 3 most populated clusters was occupied during the last 500 ns of the simulation (blue: top cluster, orange: second cluster, green: third cluster). Bottom inset: RMSF for each residue. (B) The same for simulations of Fig 2F without Mg^2+^. (C) Same for simulations of Fig 2I with Mg^2+^.
(TIF)

S3 Fig MD simulations of models with 5′-end threading. (A) Overlay of the 3D structure of the 3 most populated clusters from simulations of Fig 2G with Mg^2+^ (ions not shown for clarity). Top inset: RMSD vs time, showing when each of the 3 most populated clusters was occupied during the last 500 ns of the simulation (blue: top cluster, orange: second cluster, green: third cluster). Bottom inset: RMSF for each residue. (B) The same for simulations of Fig 2E without Mg^2+^. (C) The same for simulations of Fig 2G without Mg^2+^.
(TIF)

S4 Fig MD simulations of model with L3 threading. (A) Overlay of the 3D structure of the 3 most populated clusters from simulations of Fig 2H with Mg^2+^ (ions not shown for clarity). Top inset: RMSD vs time, showing when each of the 3 most populated clusters was occupied during the last 500 ns of the simulation (blue: top cluster, orange: second cluster, green: third cluster). Bottom inset: RMSF for each residue.
(TIF)

S1 Data PDB files for structural models. Files in.pdb format for each of the model structures presented in the manuscript are included in an archived.zip folder. The files corresponding to the structures shown in Figs 3B, 3E, 3H, 4B, 4E, 4H, 5B, 6A–6F, and 7A–7G are named Fig*NX*_1.pdb, where *N* is the figure number and *X* is the letter for the figure panel. These structures represent the most populated of the clusters found in the corresponding MD simulations. The structures of the second- and third-most populated clusters from each of the simulations are in the files named Fig*NX*_2.pdb and Fig*NX*_3.pdb, respectively.
(ZIP)
