# Supplementary material for: Modeling the structure of the frameshift-stimulatory pseudoknot in SARS-CoV-2 reveals multiple possible conformers
Source: PLoS Comput Biol. 2021 Jan 19;17(1):e1008603. doi: 10.1371/journal.pcbi.1008603 (PMC7845960; doi:10.1371/journal.pcbi.1008603)
Supplement: S1 Table — Models in red were rejected after MD simulations because of unfolded base-pairs that were inconsistent with the expected secondary structure. Models in blue were rejected before MD simulation because of a topologically knotted fold that is inconsistent with RNA. Models in green were analyzed in the main text. (DOCX) [file pcbi.1008603.s001.docx]

**S1 Table: Pseudoknot models studied**. Models in red were rejected after MD simulations because of unfolded base-pairs that were inconsistent with the expected secondary structure. Models in blue were rejected before MD simulation because of a topologically knotted fold that is inconsistent with RNA. Models in green were analyzed in the main text.

| **Monomer models** | | | | **Base-pairs unfolded** | | |
| --- | --- | --- | --- | --- | --- | --- |
| **Prediction tool** | **Fold topology, conditions** | **Rejected?** | **Rejection stage** | **S1** | **S2** | **S3** |
| FARFAR2 | unthreaded, with Mg2+ | no |  |  |  |  |
| FARFAR2 | unthreaded, without Mg2+ | no |  |  |  |  |
| FARFAR2 | 5'-threaded, with Mg2+ | no |  |  |  |  |
| FARFAR2 | 5'-threaded, without Mg2+ | no |  |  |  |  |
| FARFAR2 | L3-threaded, with Mg+ | no |  |  |  |  |
| FARFAR2 | L3-threaded, without Mg2+ | yes | after MD | 5/10 | 1/7 | 0/9 |
| SimRNA | 5'-threaded, with Mg2+ | no |  |  |  |  |
| SimRNA | 5'-threaded, without Mg2+ | yes | after MD | 4/10 | 4/7 | 1/9 |
| SimRNA | unthreaded, with Mg2+ | yes | after MD | 2/10 | 2/7 | 0/9 |
| SimRNA | unthreaded, without Mg2+ | yes | after MD | 2/10 | 2/7 | 0/9 |
| Vfold | unthreaded, with Mg2+ | no |  |  |  |  |
| Vfold | unthreaded, without Mg2+ | yes | after MD | 1/10 | 2/7 | 0/9 |
| Vfold | 5'-threaded, with Mg2+ | yes | after MD | 5/10 | 7/7 | 0/9 |
| Vfold | 5'-threaded, without Mg2+ | yes | after MD | 0/10 | 7/7 | 2/9 |
| RNAComposer | unthreaded, with Mg2+ | yes | after MD | 5/10 | 3/7 | 1/9 |
| RNAComposer | unthreaded, without Mg2+ | yes | after MD | 9/10 | 2/7 | 0/9 |
| RNAvista | unthreaded, with Mg2+ | yes | after MD | 10/10 | 1/7 | 0/9 |
| RNAvista | unthreaded, without Mg2+ | yes | after MD | 10/10 | 3/7 | 0/9 |
| RNAvista | knotted | yes | before MD | N/A | N/A | N/A |
| MC-Sym | knotted | yes | before MD | N/A | N/A | N/A |
| RNA2D3D | knotted | yes | before MD | N/A | N/A | N/A |
|  |  |  |  |  |  |  |
| **Dimer models** | | | | **Base-pairs unfolded** | | |
| **Prediction tool** | **Fold topology, conditions** | **Rejected?** | **Rejection stage** | **S1** | **S2** | **S3** |
| FARFAR2 | unthreaded/unthreaded, with Mg2+ | yes | after MD | 0/10 | 3/7 | 0/9 |
| FARFAR2 | unthreaded/unthreaded, without Mg2+ | no |  |  |  |  |
| FARFAR2 | unthreaded/5'-threaded, with Mg2+ | no |  |  |  |  |
| FARFAR2 | unthreaded/5'-threaded, without Mg2+ | no |  |  |  |  |
| FARFAR2 | unthreaded/L3-threaded, with Mg2+ | yes | after MD | 8/10 | 2/7 | 2/9 |
| FARFAR2 | unthreaded/L3-threaded, without Mg2+ | yes | after MD | 4/10 | 0/7 | 0/9 |
| FARFAR2 | 5'-threaded/L3-threaded, with Mg2+ | yes | after MD | 3/10 | 3/7 | 0/9 |
| FARFAR2 | 5'-threaded/L3-threaded, without Mg2+ | yes | after MD | 1/10 | 2/9 | 1/7 |
| FARFAR2 | 5'-threaded/5'-threaded, with Mg2+ | no |  |  |  |  |
| FARFAR2 | 5'-threaded/5'-threaded, without Mg2+ | no |  |  |  |  |
| SimRNA (manual) | 5'-threaded/5'-threaded, with Mg2+ | no |  |  |  |  |
| SimRNA (manual) | 5'-threaded/5'-threaded, without Mg2+ | no |  |  |  |  |
