# Supplementary figures and images for: Modeling the structure of the frameshift-stimulatory pseudoknot in SARS-CoV-2 reveals multiple possible conformers
Source: PLoS Comput Biol. 2021 Jan 19;17(1):e1008603. doi: 10.1371/journal.pcbi.1008603 (PMC7845960; doi:10.1371/journal.pcbi.1008603)

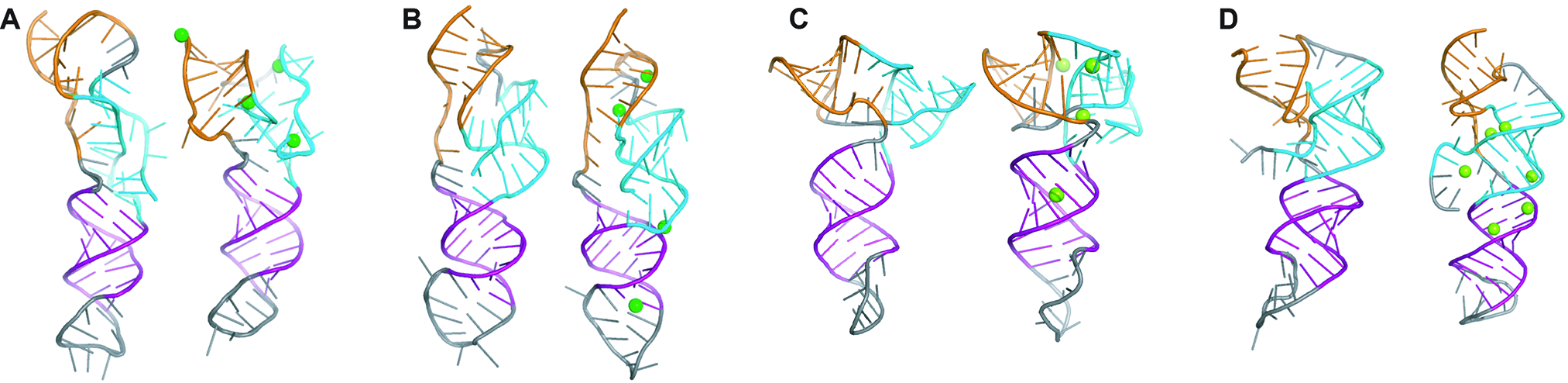

Supplement: S1 Fig — Representative structures of the most populated cluster from simulations of (A) Fig 2A, (B) Fig 2B, (C) Fig 2C, and (D) Fig 2D show significant disruption of the secondary structure. In each panel, the figure on the right is from simulations with Mg2+, that on the left is from simulations without Mg2+. (TIF) [file pcbi.1008603.s002.tif]

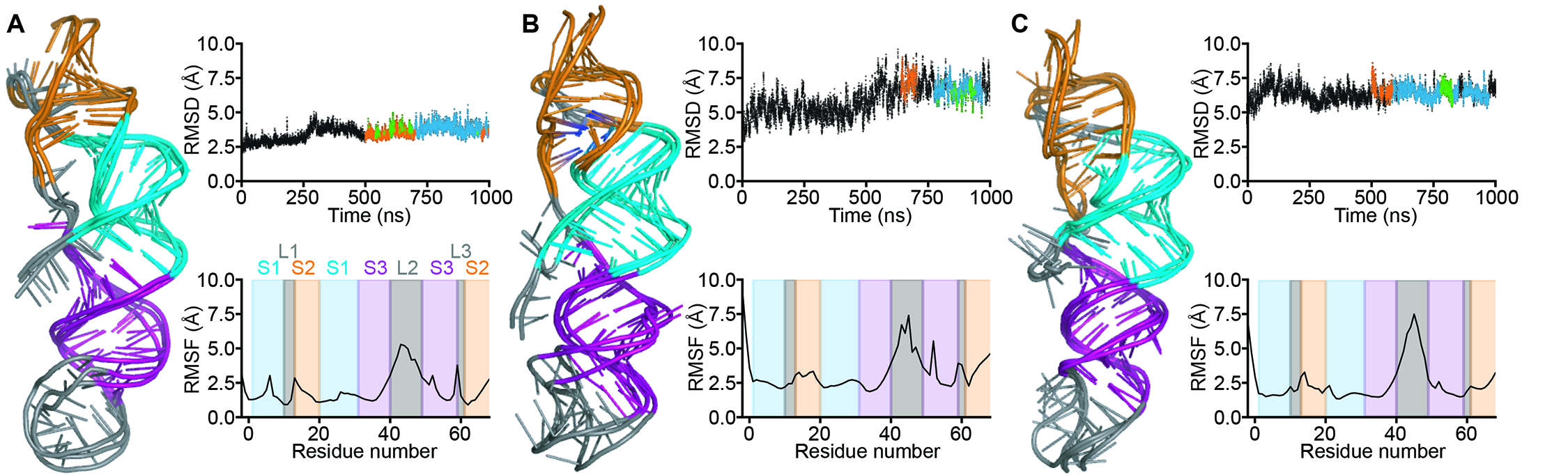

Supplement: S2 Fig — (A) Overlay of the 3D structure of the 3 most populated clusters from simulations of Fig 2F with Mg2+ (ions not shown for clarity). Top inset: RMSD vs time, showing when each of the 3 most populated clusters was occupied during the last 500 ns of the simulation (blue: top cluster, orange: second cluster, green: third cluster). Bottom inset: RMSF for each residue. (B) The same for simulations of Fig 2F without Mg2+. (C) Same for simulations of Fig 2I with Mg2+. (TIF) [file pcbi.1008603.s003.tif]

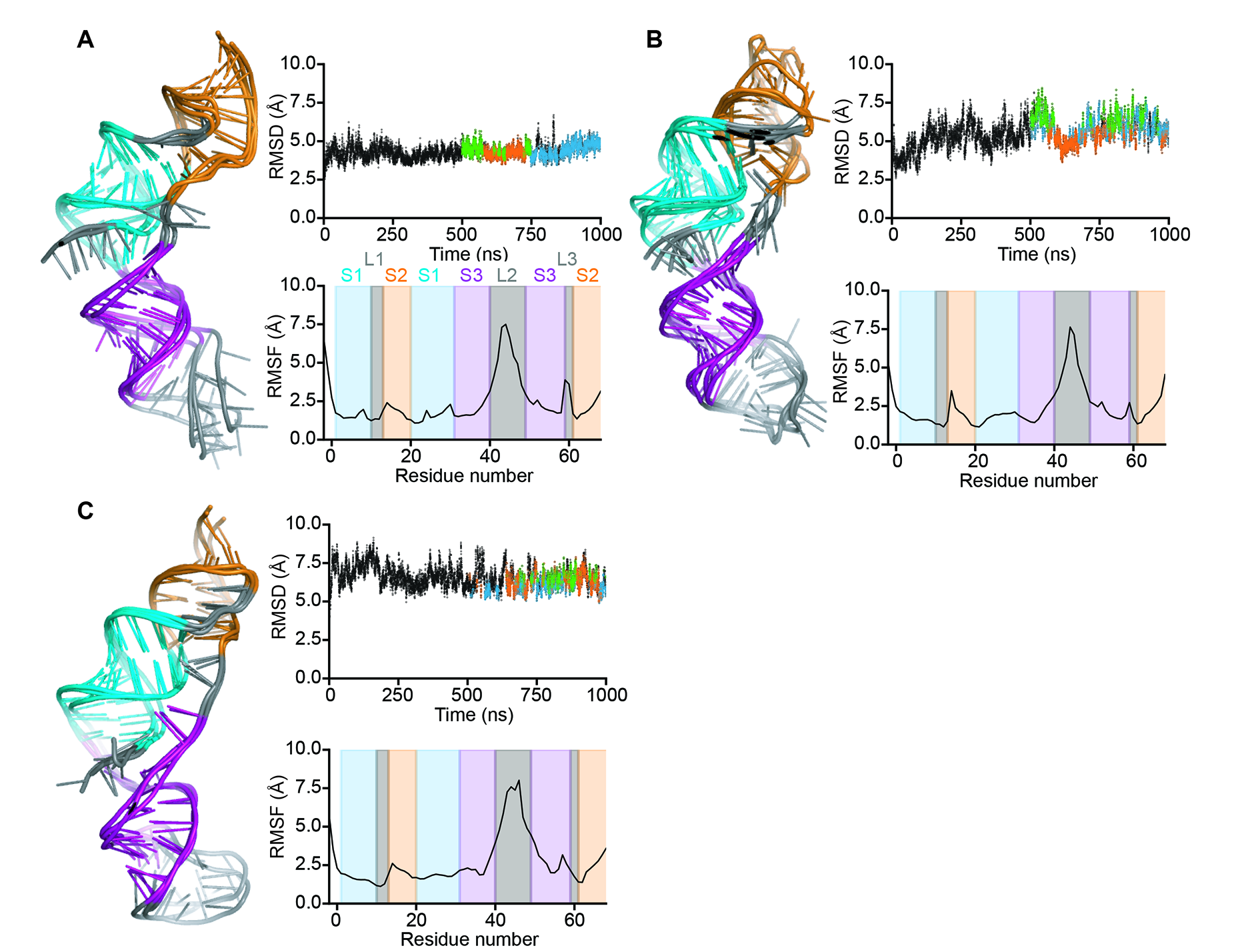

Supplement: S3 Fig — (A) Overlay of the 3D structure of the 3 most populated clusters from simulations of Fig 2G with Mg2+ (ions not shown for clarity). Top inset: RMSD vs time, showing when each of the 3 most populated clusters was occupied during the last 500 ns of the simulation (blue: top cluster, orange: second cluster, green: third cluster). Bottom inset: RMSF for each residue. (B) The same for simulations of Fig 2E without Mg2+. (C) The same for simulations of Fig 2G without Mg2+. (TIF) [file pcbi.1008603.s004.tif]

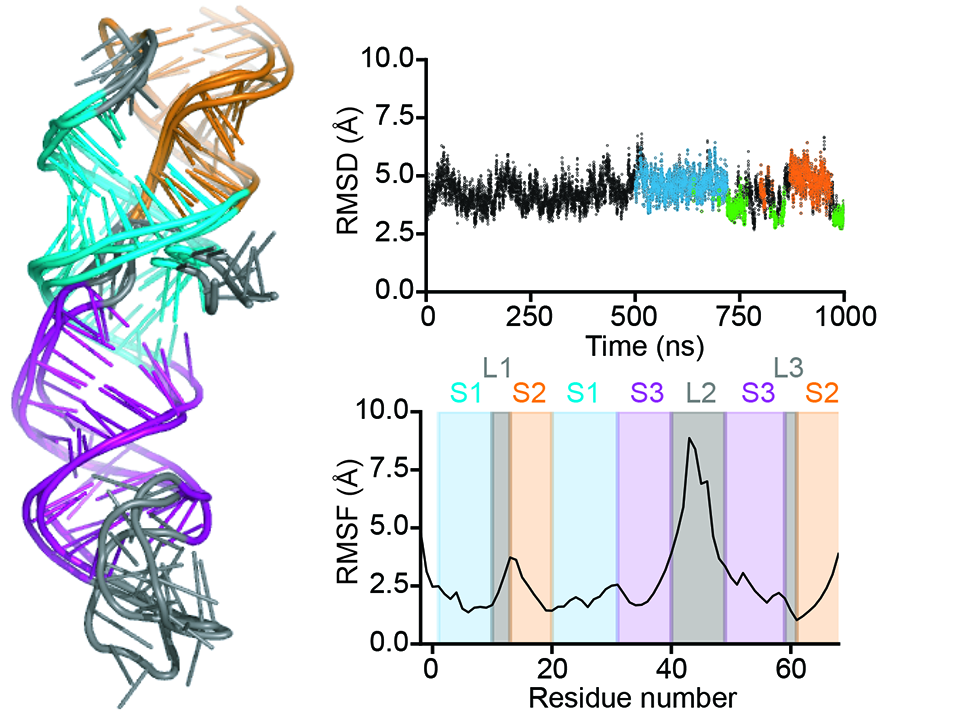

Supplement: S4 Fig — (A) Overlay of the 3D structure of the 3 most populated clusters from simulations of Fig 2H with Mg2+ (ions not shown for clarity). Top inset: RMSD vs time, showing when each of the 3 most populated clusters was occupied during the last 500 ns of the simulation (blue: top cluster, orange: second cluster, green: third cluster). Bottom inset: RMSF for each residue. (TIF) [file pcbi.1008603.s005.tif]
